# Conjunctival lymphoma during pregnancy: a case report

**DOI:** 10.1186/s12886-017-0518-z

**Published:** 2017-07-27

**Authors:** Sherine Jue Ong, Shih-Ming Jung, Hsin-Chiung Lin

**Affiliations:** 1 Department of Ophthalmology, Chang Gung Memorial Hospital, Chang Gung University, Keelung, Taiwan; 2 Department of Pathology, Chang Gung Memorial Hospital, Chang Gung University, Taoyuan, Taiwan; 3 Department of Ophthalmology, Chang Gung Memorial Hospital and University, #5, Fu-Hsing Street, Kweishan, Taoyuan, 333 Taiwan

**Keywords:** Pregnancy and conjunctival lymphoma, Case report

## Abstract

**Background:**

To present a case of conjunctival lymphoma in a young woman complicated by pregnancy.

**Case presentation:**

A 38-year-old previously healthy woman presented with a 2-year history of progressive right blepharoptosis. Giant papillomatous sessile masses were identified in the upper and lower fornix bilaterally and involved the tarsus of the right upper lid. The remaining ophthalmic examination was unremarkable. Histopathology and immunohistochemistry showed mucosa-associated lymphoid tissue (MALT) lymphoma with immunoglobulin kappa monotype. Further workup showed no evidence of systemic lymphoma or orbital involvement.

**Conclusions:**

Partial regrowth of conjunctival lymphoma occurred 6 months after excision and the MALT lymphoma remained indolent during the course of her pregnancy without radiotherapy.

## Background

Lymphomas that occur outside of lymph nodes or the spleen are defined as extranodal and account for about 24% to 48% of lymphomas. Mucosa-associated lymphoid tissue (MALT) lymphoma (also known as extranodal marginal zone B-cell lymphoma of mucosa-associated lymphoid tissue) is the predominant histopathological type of malignant lymphoma of the conjunctiva. It presents primarily in female patients in their late 60s and is often localized and of indolent clinical behavior [1–8]. It does occur in young adult or children [9–11]. “Salmon patch” is the typical clinical presentation of conjunctival lymphoma, and, unlike orbital lymphoma, such conjunctival lesions rarely present as blepharoptosis [2, 12]. Here we report a case of conjunctival lymphoma in a young woman complicated by pregnancy.

## Case presentation

**Fig. 1:**
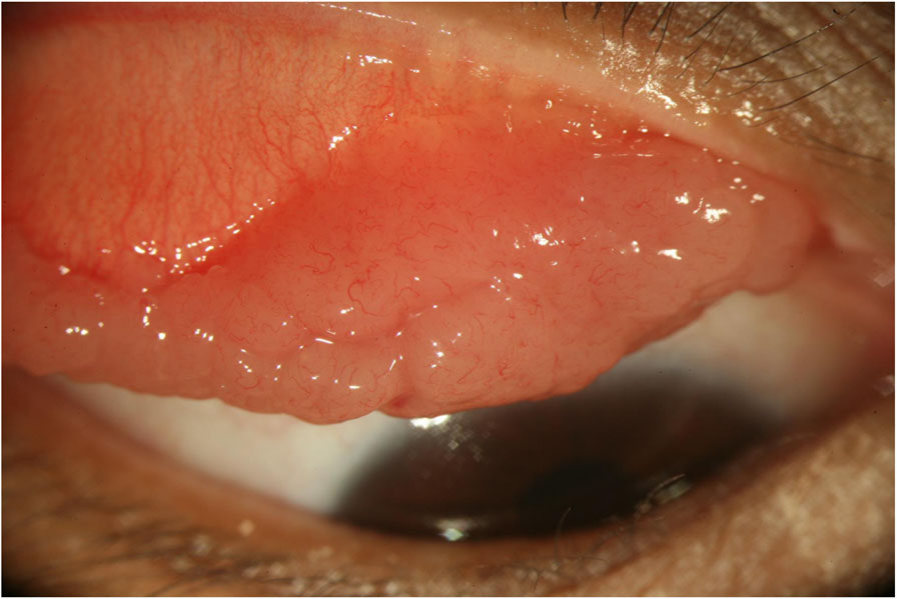
Clinical photograph of the right upper lid MALT lymphoma causing blepharoptosis

The study protocol adheres to the tenets of the Declaration of Helsinki, and was approved by the Human Research Ethics Committee at Chang Gung Memorial hospital, Taiwan (No. 100-2022B). A 38-year-old woman visited the Chang Gung Memorial Hospital in March 2010 complaining of progressive painless right blepharoptosis over a 2-year period. She had no relevant medical history. The initial ocular examination showed that her best-corrected visual acuity was 20/20 in each eye, and her eye movements were full in both eyes. The anterior and posterior segments of both eyes were normal. A giant papillomatous mass was identified in the entire superior tarsal conjunctiva of the right eye (Fig. 1) but did not cause symptoms other than progressive ptosis. In addition, multinodular conjunctival tumors were also found and occupied one-third to one-half of the left upper and lower fornix in both eyes. The remaining ophthalmic examination was unremarkable.

**Fig. 2:**
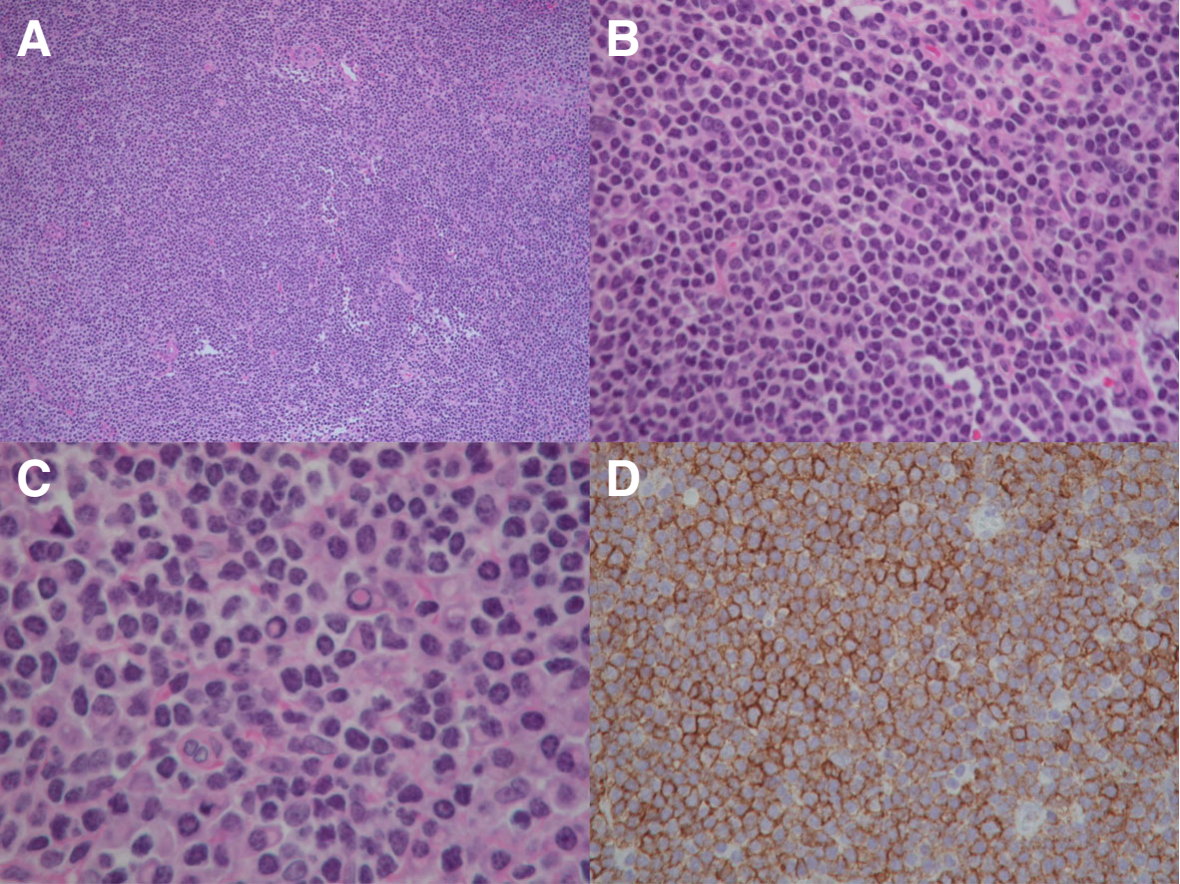
**a** Low*-*power magnification of the conjunctival tumor, demonstrating an infiltration of small lymphoid cells (hematoxylin and eosin stain, original magnification ×20). **b** Heterogeneous cells with minimal cytoplasm and prominent nuclei and nucleoli (hematoxylin and eosin stain, original magnification ×100). **c** MALT lymphoma demonstrates Dutcher bodies with nuclear pseudoinclusions. (hematoxylin and eosin stain, original magnification ×400). **d** Tumor cells are immunopositivity for CD20 (original magnification ×200)

A complete excisional biopsy was performed in the right upper and lower eyelids, which relieved the severity of ptosis in the right eye. The pathology examination using hematoxylin-eosin stain and immunohistochemistry studies revealed extranodal marginal zone lymphoma of mucosa-associated lymphoid tissue (MALT lymphoma) with immunoglobulin kappa monotype (Fig. 2). The Ki-67 proliferation index was low. The patient was referred to a hematologist for staging investigations including complete blood count, hepatic enzymes including lactate dehydrogenase, and orbit, thorax, and abdominal computed tomography (CT) scans, as well as bone marrow examination. CT demonstrated conjunctival thickening and no other organ involvement; results of the bone marrow study were normal. Ann Arbor stage IE primary ocular adnexal lymphoma was diagnosed, and, accordingly, local radiation therapy was suggested [13]. The patient chose observation of the lesion instead of radiation therapy because she was concerned about the dry eye complication after radiation therapy and she was planning to become pregnant. Focal regrowth in right lower fornix of MALT lymphoma was noted 6 month after excision. She delivered a baby 16 months after the excisional biopsy and was followed up in our clinic for 5 years with no further progression of ptosis, there was no change in her health.

## Discussion

Extranodal lymphomas can occur in a variety of organs, including the conjunctiva, orbit, salivary glands, skin, thyroid, lungs, stomach, and intestines. Ocular adnexal involvement occurs in <10% of extranodal lymphomas [14]. Conjunctival MALT lymphoma can have a variety of presentations in the periocular region. Diagnosis is often made on the basis of the typical clinical presentation of a “salmon patch” conjunctival mass and pathologic evaluation following a tissue biopsy. To the best of our knowledge, there are no reports describing conjunctival lymphoma during pregnancy.

Conjunctival lymphomas can masquerade as chronic conjunctivitis that does not respond to various treatments [15]. Strauss et al. reported a 32-year-old man who presented with follicular-like palpebral conjunctivitis with unilateral blepharoptosis that proved to be a B-cell lymphoma [12]. Shields et al. reported a series of 117 cases of conjunctival lymphoid tumors. In the majority (30%) of cases, mass was first noted by the patient, followed by irritation (29%) and ptosis (8%), and none of the tumors in this series were located in the tarsus [2].

Here we report a case of tarsal conjunctival lymphoma for which blepharoptosis was the sole manifestation. MALT lymphomas occur in patients of all ages but are most common in patients aged 50–70 years. The differential diagnosis includes the following: Conjunctival papilloma typically presents with a pink sessile or pedunculated configuration; conjunctival amyloidosis can appear as a yellow to pink diffuse mass; and chronic papillomatous or follicular conjunctivitis and metastatic tumors can appear as multiple elevated fleshy pink vascularized tumors that may resemble conjunctival lymphoma clinically. Tumor biopsy is critical to differentiate conjunctival tumors.

The most common ocular site for lymphoma involvement is the orbit. McKelvie et al. reported a series of 73 patients with lymphoproliferative lesions of the ocular adnexa, disease of the orbit and lacrimal gland comprised 74.3%, and conjunctival disease was noted in 17% in their series [16]. There may be clinical clues about the location of ocular lymphoma suggestive of the presence or development of systemic lymphoma. Knowles et al. reported that conjunctival lymphoma was associated with a lower incidence of systemic lymphoma (20%) compared with orbit (35%) or eyelid (70%) involvement [17]. Conjunctival lymphoma occurred most frequently in the fornix and midbulbar area, as reported by Shields et al., and bilateral lesions were noted in 10%–17% of the patients [2]. Systemic lymphoma occurred more frequently at extralimbal sites and with multiple tumors, about 38% of patients develop systemic lymphoma in 5 years, and thus longer follow-up is necessary [2].

Our patient was relatively young at the onset of lymphoma, and systematic workup showed no other organ involvement. Immunohistochemical study showed positivity for CD20 with the absence of staining for CD3 lymphocytes, along with a monotypic expression of kappa light chains. In patients with stage IE ocular adnexal lymphomas, localized radiotherapy is the treatment of choice, and surgery alone is not recommended because of the high recurrence rate [1, 2, 6, 18–20]. Intraocular extension is rare [21]. In selected patients, the disorder can be managed with observation alone [22–24]. The patient had risk factors for systemic metastasis because the lymphoma was located in the fornix and tarsus, as well as multiple conjunctival tumors. She declined radiotherapy because of her child-bearing plan, partial regrowth of right lower fornix conjunctival lesion of MALT lymphoma was noted 6 month after excision. The second biopsy in this patient demonstrated the same histopathological type of the diagnosis. She delivered a healthy baby 16 months after conjunctival tumor excision, and the MALT lymphoma remained indolent during her pregnancy.

## Conclusion

Conjunctival MALT lymphoma during pregnancy is uncommon. When making treatment decisions for cancer during pregnancy, it is important to consider the best treatment options for the pregnant woman balanced against the possible risks to the growing baby. Surgery poses little risk to the growing baby and is considered the safest cancer treatment during pregnancy. Radiation therapy can harm the fetus in all trimesters; physicians generally avoid using this treatment during pregnancy. The risks to the baby depend on the dose of radiation and the area of body being treated. In our case, the conjunctival lymphoma remained latent during her pregnancy; there was no systemic metastasis in 5 years with excision therapy alone. More information is needed for the effect of pregnancy on the clinical course of conjunctival lymphoma.

## Abbreviations

CT: Computed tomography
MALT: Mucosa-associated lymphoid tissue
